# Insights into Complex Compounds of Ampicillin: Potentiometric and Spectroscopic Studies

**DOI:** 10.3390/ijms26157605

**Published:** 2025-08-06

**Authors:** Justyna Frymark, Michał Zabiszak, Jakub Grajewski, Bartosz Tylkowski, Renata Jastrzab

**Affiliations:** 1 Faculty of Chemistry, Adam Mickiewicz University in Poznan, Uniwersytetu Poznańskiego 8, 61-614 Poznan, Poland; zabiszakm@amu.edu.pl (M.Z.); jakub.grajewski@amu.edu.pl (J.G.); renatad@amu.edu.pl (R.J.); 2 Eurecat, Centre Tecnològic de Catalunya, Unitat de Tecnologia Química, Marcellí Domingo 2, 43007 Tarragona, Spain; bartosz.tylkowski@eurecat.org; 3 Department of Clinical Neuropsychology, Faculty of Health Science, Ludwik Rydygier Collegium Medicum in Bydgoszcz, Nicolaus Copernicus University in Torun, ul. Sklodowskiej Curie 9, 85-094 Bydgoszcz, Poland

**Keywords:** metal ion complexes, ampicillin, complex compounds, spectroscopy, potentiometric measurements

## Abstract

Metal ions, including Mg(II), Ca(II), Sr(II), Co(II), Ni(II), Cu(II), Nd(III), Eu(III), and Tb(III), were investigated in binary systems alongside ampicillin at molar ratios of 1:1 and 1:2. These investigations were carried out in aqueous solutions, and the formation of complexes was verified through meticulous computational analysis. Detailed stability constants for the formed complexes and equilibrium constants for the involved reactions were meticulously determined. Furthermore, a comprehensive examination of the impact of ligand concentration on the configuration of the central metal atom’s coordination sphere was conducted. This investigation was complemented by spectroscopic measurements, which effectively confirmed the observed changes in the coordination sphere of the metal ions.

## 1. Introduction

**Figure 1:**
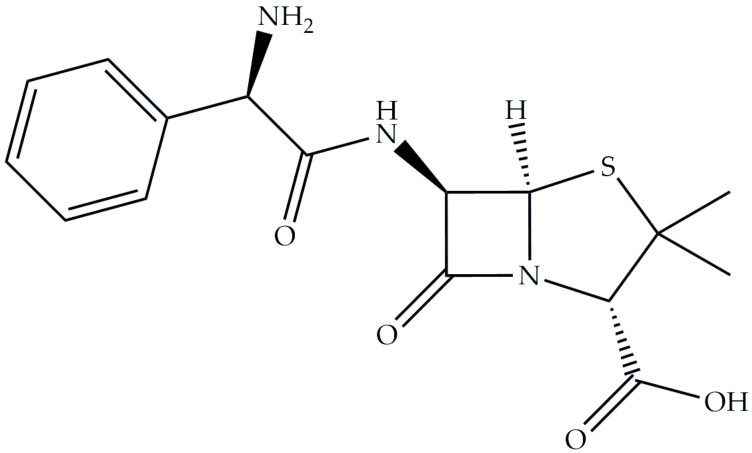
Formula of ampicillin.

**Figure 2:**
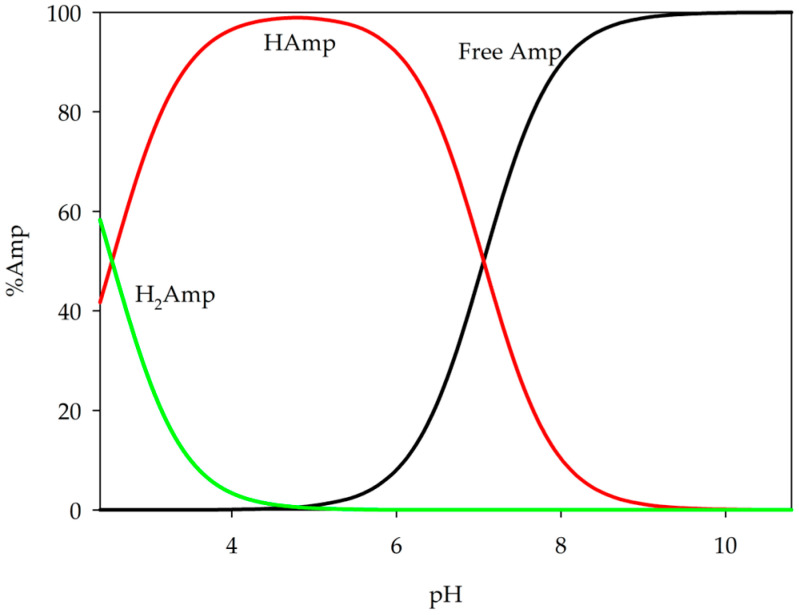
Distribution diagram of ampicillin.

Ampicillin (Amp) (Figure 1), (2S,5R,6R)-6-([(2R)-2-amino-2-phenylacetyl]amino)-3,3-dimethyl-7-oxo-4-thia-1-azabicyclo [3.2.0]heptane-2-carboxylic acid, is a β-lactam antibiotic belonging to the group of aminopenicillins, derivatives of penicillin, and has been widely recognized for its efficacy in the treatment of bacterial infections [1,2,3,4,5,6]. The effect of this antibiotic is to combat bacterial infections by inhibiting the production of bacterial cell walls, leading to their disintegration. Its mechanism involves blocking bacterial cell wall synthesis by binding to penicillin-binding proteins, which ultimately compromises the structure of the bacterial cell wall and leads to cell lysis [7,8]. Ampicillin, a semisynthetic derivative of penicillin, is a cornerstone in the arsenal of antibiotics because of its broad-spectrum activity against a wide range of bacterial pathogens. It is often recommended for respiratory and urinary tract infections, specific forms of meningitis, and similar diseases [5,9,10,11]. Adherence to the prescribed dose and completion of the duration of treatment are crucial to maximize effectiveness and decrease the development of potential antibiotic resistance [12,13,14,15,16].

However, despite its early effectiveness, extensive utilization and incorrect use of ampicillin have resulted in increased antimicrobial resistance, which presents a notable concern for public health on a global scale [17,18,19,20,21]. Bacteria have devised various strategies to overcome the impact of antibiotics. Among these is the creation of β-lactamase enzymes that break down the β-lactam ring of ampicillin, making it incapable of effectively fighting bacterial infections. These advances underscore the importance of judicious antibiotic use and the exploration of substitute therapies for microbial infections [22,23,24,25,26].

In addition to the challenge of antimicrobial resistance, recent research has focused on exploring the complex interactions of ampicillin with metal ions. Metal ions play a diverse role in biological systems, influencing enzyme activity, redox reactions, and cellular processes. Furthermore, the coordination of ampicillin with metal ions can affect its pharmacological properties by altering its stability, bioavailability, and antimicrobial activity [27,28,29,30,31,32].

The study of the interactions between ampicillin and metal ions may be crucial in pharmacology, providing valuable information on how this antibiotic behaves in living organisms or in the environment. Investigating the impact of metal ions on the effectiveness of ampicillin could lead to new approaches to improve its efficacy and address resistance mechanisms, ultimately significantly advancing antibiotic treatment options [33,34,35,36]. We selected s-block (Mg(II), Ca(II), Sr(II)) and d-block (Co(II), Ni(II), Cu(II)) ions that are both essential in human physiology and commonly encountered in environmental matrices. The f-block lanthanides (Nd(III), Eu(III), Tb(III)) were chosen due to their growing use in luminescent probes and theranostic applications.

In this paper, we present the results of potentiometric and spectroscopic studies on complex formation in systems involving ampicillin and selected metal ions. By gaining insight into molecular-level interactions and exploring the structural and functional characteristics of complexes formed between ampicillin and metal ions, we aim to improve our understanding of antibiotic mechanisms and lay the groundwork for the advancement of new antimicrobial treatments for addressing AMR while improving antibiotic therapy.

## 2. Results and Discussion

This research involved the examination of binary systems comprising ampicillin and metal ions using potentiometric titration and spectroscopy methods. The first stage of this experiment was to determine successive protonation constants for ampicillin, which were found to be log*β* = 7.06 for H(Amp) and log*β* = 9.61 for the form H_2_(Amp) (p*K*a_1_ = 2.55; p*K*a_2_ = 7.06). Literature p*K*a values for ampicillin are 2.54 and 7.30 [27,31]; however, our values differ due to the use of a different temperature and ionic strength (*I* = 0.10 mol·dm^−3^ KNO_3_). Analysis of these values of ampicillin protonation constants revealed deprotonation of the carboxyl group at relatively low pH values, followed by deprotonation of the amino group at pH values above 5.0 (Figure 2). Subsequently, the obtained values of the protonation constant of ampicillin and the hydrolysis constant of metal ions previously determined were used as a set of data for theoretical computations [36,37,38,39].

### 2.1. Equilibrium Study of S-Block Elements

A series of equimolar titrations and a two-fold excess of ampicillin were performed. Then, on the basis of the data obtained, the overall stability constants (log*β*) of the complexes formed in the system and the equilibrium constants of the formation of the complex (log*K_e_*) were determined, as shown in Table 1.

**Figure 3:**
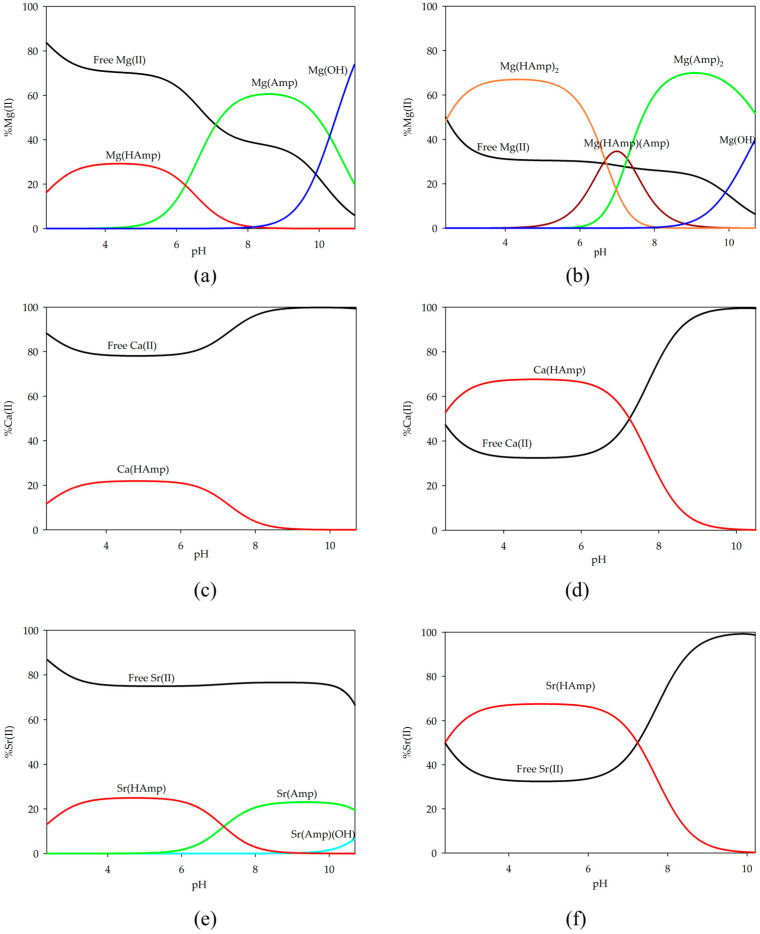
Distribution diagrams for the systems studied: (**a**) Mg(II)/ampicillin (1:1 ratio); (**b**) Mg(II)/ampicillin (1:2 ratio); (**c**) Ca(II)/ampicillin (1:1 ratio); (**d**) Ca(II)/ampicillin (1:2 ratio); (**e**) Sr(II)/ampicillin (1:1 ratio); (**f**) Sr(II)/ampicillin (1:2 ratio).

The distribution diagrams of the complex forms were plotted using the obtained stability constant values (Figure 3). In the equimolar system, only a small amount of the ions participate in the complexation process and form complexes with one molecule of ampicillin in the inner coordination sphere. At a low pH value, when the complexation process begins and the ampicillin molecule becomes partially deprotonated, the M(HAmp) form is observed as the only form in each of the tested systems for the s-block elements. For the system with magnesium ions, as the pH increases within the range of 7.0 to 9.8, the Mg(Amp) complex becomes dominant and binds over 60% of the magnesium ions.

In a ligand-excess system involving magnesium ions, various forms with two molecules of ampicillin were observed. Initially, both molecules are partially deprotonated; then, within a narrow pH range (6.7–7.2), fully and partially deprotonated molecular forms dominate until pH 7.2, where Mg(Amp)_2_ becomes dominant up to pH 10.7.

In the case of calcium ions, the Ca(HAmp) form is the sole species present in both the equimolar system and the two-fold excess of ampicillin. In the equimolar system, this form binds only 20% of the ions, whereas, with an excess of ampicillin, 65% of the studied ligand participates in metal coordination. A similar situation is observed for strontium ions, where the same proportion of ions engage in complexation processes. However, in the equimolar system, a complex with a fully deprotonated ligand was also observed for strontium ions, although only approximately 20% of the metal ions participated in coordination. In addition, the formation of a hydroxy complex was observed in this system.

### 2.2. Equilibrium Study of D-Block Elements

**Figure 4:**
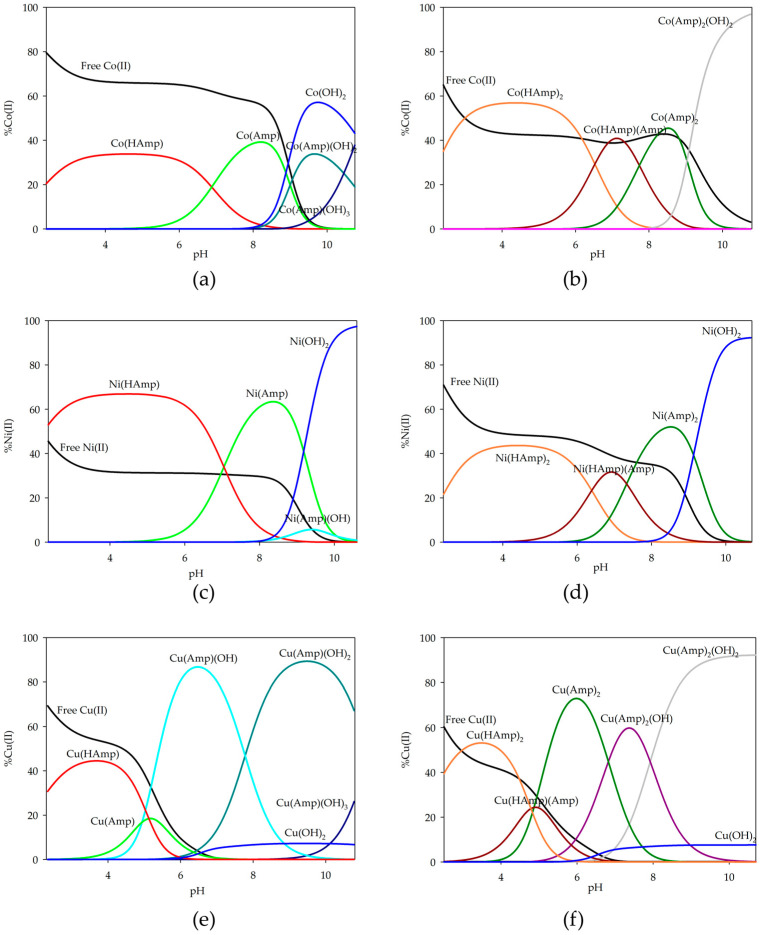
Distribution diagrams for the systems studied: (**a**) Co(II)/ampicillin (1:1 ratio); (**b**) Co(II)/ampicillin (1:2 ratio); (**c**) Ni(II)/ampicillin (1:1 ratio); (**d**) Ni(II)/ampicillin (1:2 ratio); (**e**) Cu(II)/ampicillin (1:1 ratio); (**f**) Cu(II)/ampicillin (1:2 ratio).

For d-block metal ions (Co(II), Ni(II), Cu(II)), a series of potentiometric titrations were performed, and the overall stability constants (log*β*) and the equilibrium constants of formation (log*K_e_*) were calculated (Table 2). Distribution diagrams were plotted as illustrated in Figure 4. In the case of equilibrium studies with d-block metal ions, the presence of a larger number of various complex forms in the systems can be observed. In the equimolar systems, only complexes with one coordinated ampicillin molecule were observed, while an excess of antibiotic led to forms with two ampicillin molecules in the inner coordination sphere of the metal ion. Notable differences compared to systems with s-block ions include the presence of a greater number of complexes and a higher percentage of ion participation in the complexation process.

In the equimolar system, the complex with a partially deprotonated form of ampicillin occurs at lower pH values. For cobalt(II) ions, the M(HAmp) form binds about 40% of the ions; for nickel(II) ions, it is the dominant form and binds over 60% of the ions, while for copper(II), it binds about 44%. As the pH value increases, the predominant form in the system becomes the M(Amp) form. At higher pH values, hydroxyl complexes begin to form M(Amp)(OH)_x_, where x = 1–3. In the systems with cobalt and nickel ions, they are not dominant due to the dominance of metal hydroxides. However, for the copper(II) ion system, the dominant is M(Amp)(OH)_2_ binding about 90% of the present ions.

In systems with an excess of ampicillin, forms with two ligand molecules coordinated to the inner coordination sphere were identified. Similarly to what was observed in magnesium-complexed ampicillin systems at lower pH values, complexation begins with the formation of a complex with a two-molecule incompletely deprotonated ligand, which binds approximately half of the available metal ions. At around pH 7.0, when the fully deprotonated ligand is present in the system, the M(HAmp)(Amp) complex obtains the maximum concentration.

It should be noted that for both copper-based systems, ligand deprotonation occurs at lower pH values, causing the Cu(HAmp)(Amp) species to dominate at lower pH compared to other metals. The dominant form in the systems and reaching its maximum concentration at pH around 7.0 is the Cu(Amp)_2_ form. At pH above 8.0, for copper and cobalt systems, dihydroxyl complexes appear, binding almost 90% of the ions present in the solution. However, under the same conditions for the system with nickel ions, the dominant form is hydroxide.

### 2.3. Equilibrium Study of F-Block Elements

**Figure 5:**
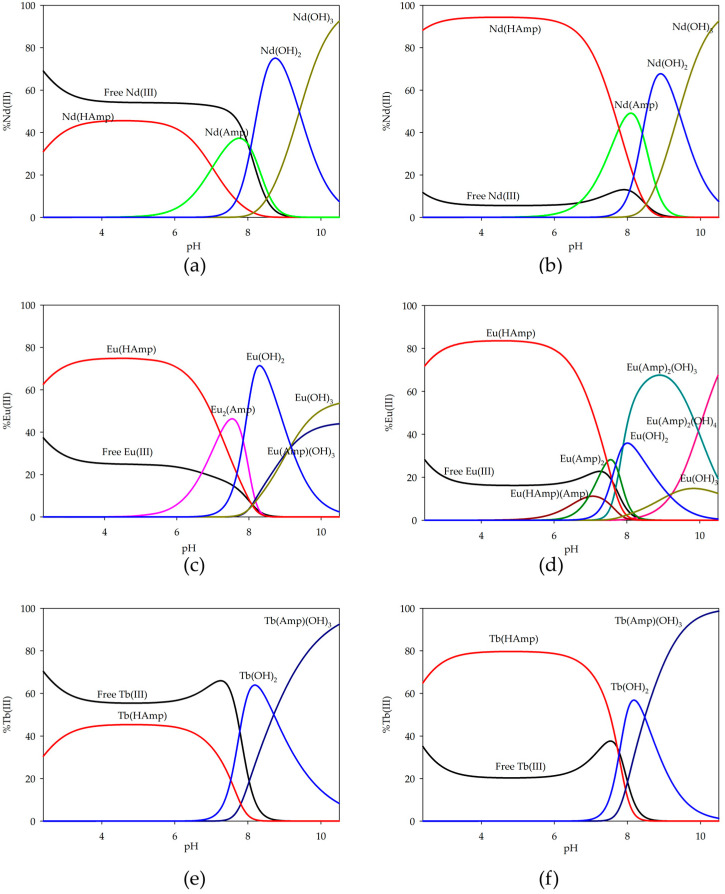
Distribution diagrams for the systems studied: (**a**) Nd(III)/ampicillin (1:1 ratio); (**b**) Nd(III)/ampicillin (1:2 ratio); (**c**) Eu(III)/ampicillin (1:1 ratio); (**d**) Eu(III)/ampicillin (1:2 ratio); (**e**) Tb(III)/ampicillin (1:1 ratio); (**f**) Tb(III)/ampicillin (1:2 ratio).

The complexation process for f-block metals was also examined. The overall stability constants (log*β*) and the equilibrium constants of formation (log*K_e_*) of the complexes are presented in Table 3, and the distribution diagrams are shown in Figure 5.

In f-block metal ion systems, a different coordination method for europium ions is observed. In equimolar systems with neodymium ions, there is a partially deprotonated ampicillin form that binds about 40% of metal ions. As the pH increases, only the Nd(Amp) form occurs. However, neither of these forms dominates the system. Above pH 8.0, only neodymium hydroxides occur. Similar forms are present in excess ampicillin systems, with the notable difference being the significant quantitative participation of neodymium ions in the complexation process. The Nd(HAmp) form binds over 90% of the ions in solution, and the Nd(Amp) form binds approximately 60%. Subsequently, as seen in equimolar systems, hydroxides are predominant.

In equimolar systems with europium ions, the initial form to appear is similar to that of the neodymium system. Subsequently, the next form that occurs involves two europium ions coordinated to a single ampicillin molecule. This particular form reaches its peak concentration at around pH 7.3. Following this, there are formations of europium hydroxides and a hydroxy complex; however, these do not dominate within the system. However, in systems with excess ampicillin, there is a wider range of forms of complex compounds. At lower pH values, Eu(HAmp) initially appears, which binds about 80% of the europium ions. Then, due to the deprotonation of ampicillin and the involvement of another molecule in the coordination species containing two ampicillin molecules, a high pH level above 8.0 is reached in the case of hydroxyl complexes and the corresponding europium hydroxides, while these also formed, but without domination, as observed in other systems.

In both systems, terbium ions exist in the same forms but with varying degrees of involvement in the complexation process. In an equimolar system, around 50% of terbium ions bind to the initial Tb(HAmp) form, while this increases to about 80% in a system with a two-fold excess. Subsequently, terbium hydroxide is formed, and from pH 9.0 onwards, the predominant form present in solution is the hydroxy complex Tb(Amp)(OH)_3_, and almost 100% of the metal ions are involved in coordination at pH 11.0.

All spectroscopic measurements (UV–Vis, FTIR, EPR, CD, luminescence) were performed in solution under conditions where multiple species are known to coexist in equilibrium, as determined by potentiometric titrations. The formation of coordination compounds in systems containing metal ions was confirmed by spectroscopic techniques. These measurements were carried out at selected pH values, based on speciation diagrams, to ensure the highest possible proportion of the desired complex form in solution.

### 2.4. UV–Vis Spectroscopy

**Figure 6:**
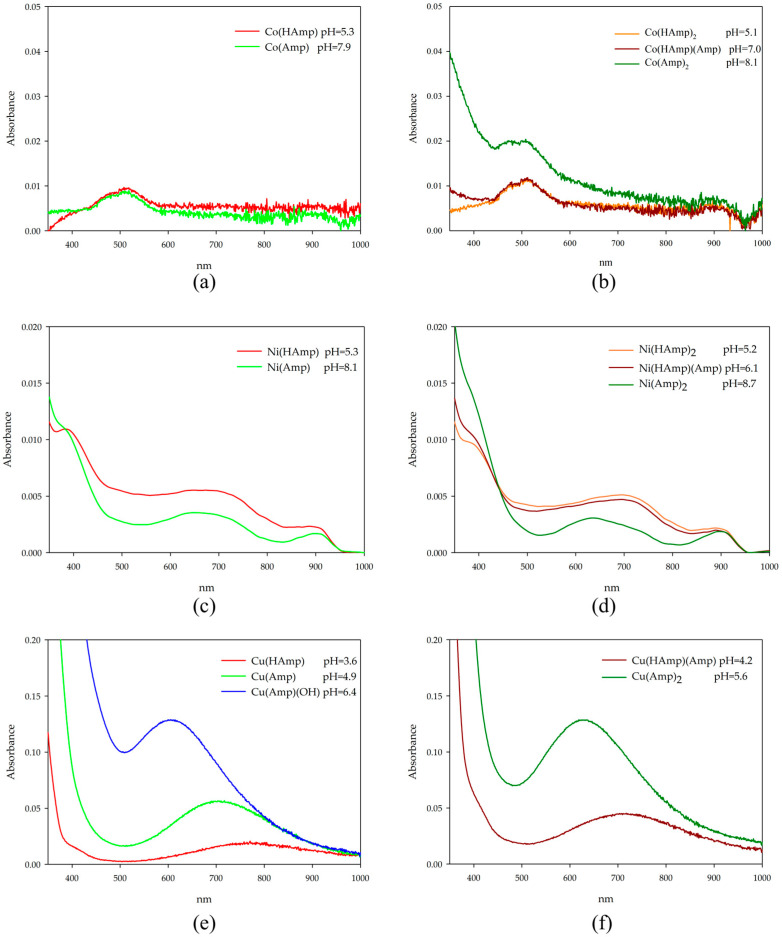
UV–Vis absorption spectra of (**a**) Co(II)/ampicillin (1:1 ratio); (**b**) Co(II)/ampicillin (1:2 ratio); (**c**) Ni(II)/ampicillin (1:1 ratio); (**d**) Ni(II)/ampicillin (1:2 ratio); (**e**) Cu(II)/ampicillin (1:1 ratio); (**f**) Cu(II)/ampicillin (1:2 ratio).

d-electron elements, such as cobalt(II), nickel(II), and copper(II), are characterized by the presence of electrons in the d subshell. Electron transitions within this subshell (e.g., d-d transitions) cause absorption of radiation in the visible and ultraviolet range. These transitions are often well defined and lead to characteristic absorption bands in ultraviolet-visible (UV–Vis) spectra. The confirmation of the coordination of the transition metal ions studied with ampicillin is provided by the characteristic shifts and changes in the absorption spectra. The observed changes are shown in Figure 6, and the spectroscopic parameters of the systems studied are placed in Table 4. Due to the solubility limits of the studied systems, the absorbance values remained below 0.1 AU in several cases. As a result, the molar absorptivity coefficients reported in Table 4 should be considered as approximate values. Changes in absorbance are interpreted cautiously and serve as qualitative indicators of coordination changes, supported by complementary data (e.g., EPR, FTIR).

In systems with cobalt ions, the d-d parameters suggest a similar coordination in the equimolar and two-fold excess of ligand systems. To account for the presence of uncomplexed Co^2+^ in the UV–Vis spectra of metal–ligand systems, the spectrum of free Co^2+^ (recorded under identical conditions) was subtracted from the mixture spectrum. The subtraction was qualitative and did not involve scaling by molar absorptivity or estimated proportions, but speciation data were considered during the interpretation to identify the dominant absorbing species. In this case of the CoL and CoL_2_ complex, a slight shift towards higher wavelengths was observed, indicating a change in the metal ions’ internal coordination sphere. For hydroxy complexes, spectral recording was impossible due to precipitate formation. Although identical concentrations were used in potentiometric studies, the dynamic potentiometric cell conditions (slow titrant dosing, continuous stirring, inert atmosphere) prevented visible M(OH)_2_ precipitation that was encountered in static cuvette measurements. For nickel-ion complexes, slight shifts toward longer wavelengths were observed along with a decrease in the molar absorption coefficient for the maximum wavelength as pH increased. In copper ion systems, the absorbance shifted toward lower wavelengths, and there was an increase in the molar absorption coefficient for the maximum wavelength as the pH increased. UV–Vis measurements at higher pH values were not performed due to precipitate formation. These results overall imply changes in the internal coordination sphere of the metal ion with increasing pH.

### 2.5. EPR Spectroscopy

**Figure 7:**
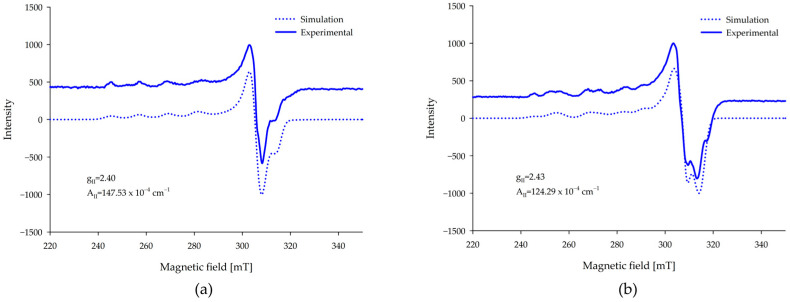
Experimental and simulated EPR spectra of (**a**) Cu(HAmp); (**b**) Cu(HAmp)_2_.

EPR measurements confirmed that only monomorphic forms occurred in systems with copper(II) ions. At low pH values, characteristic spectra for copper ions were observed (Figure 7). In the equimolar system, the form of Cu(HAmp) indicates coordination of the oxygen atom from partially deprotonated ampicillin. However, in the system with a twofold excess of ampicillin, Cu(HAmp)_2_ is formed at low pH and also indicates coordination by oxygen atoms. As the pH increases, there is an observable shift toward higher gII values in the EPR spectra, indicating a change in the coordination environment around the copper (II) ions. The spectrum of the Cu(II)-Amp system at pH 4.0 (Figure 7b) indicates the presence of multiple species in equilibrium, as evidenced by more than four hyperfine lines in the parallel region. The g∥ and A∥ values reported correspond to the most intense signal components. Although the coordination mode is tentatively assigned to involve oxygen donor atoms based on the position of these parameters in the Peisach–Blumberg diagram [40].

### 2.6. Luminescence Spectroscopy

**Figure 8:**
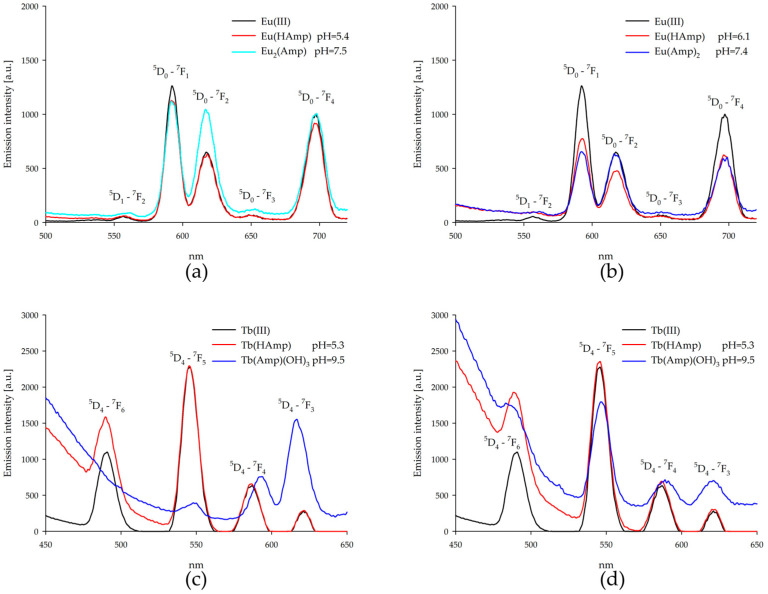
Emission spectra of the systems: (**a**) Eu(III)/ampicillin (1:1 ratio); (**b**) Eu(III)/ampicillin (1:2 ratio); (**c**) Tb(III)/ampicillin (1:1 ratio); (**d**) Tb(III)/ampicillin (1:2 ratio).

Drawing on the fundamental concepts and applications of lanthanide luminescence [41,42], emission spectra were measured for ampicillin binary systems with europium(III) ions and terbium(III) ions at the pH of the dominant complex forms (Figure 8). For europium(III) ion complexes with higher pH values, no emission measurements were carried out due to the formation of europium(III) hydroxide precipitate. When europium (III) ions were excited, emissions from the ^5^D_0_ state to ^7^F_j_ (j = 1, 2, 3, 4) levels and from the ^5^D_1_ state to the ^7^F_2_ level were observed. The emission band was quenched for formed complexes, while a two-fold increase in emission of the ^5^D_0_-^7^F_2_ band was observed for the Eu_2_(Amp) complex. Additionally, when a two-fold excess of ampicillin was used in one system, narrower emission bands were observed from the excited nondegenerate state.

Excitation of terbium(III) ions resulted in emission from the excited nondegenerate state ^5^D_4_ to the energy level of ^7^F_j_ (j = 3, 4, 5, 6) of the ground state. The observed bands showed an increase in intensity and band shifts for complexes with a partially deprotonated ampicillin molecule, Tb(HAmp), and the equimolar system of the hydroxy complex (Tb(Amp)(OH)_3_). Furthermore, there was a sharp decrease in the intensity of the ^5^D_4_ -^7^F_5_ band. In cases with excess ampicillin, this band was also extinguished, but to a lesser extent, while showing only a small increase in intensity. The baseline rise at high pH likely reflects light scattering by incipient Tb(OH)_3_ precipitate, observed as faint turbidity. Emission intensities should thus be treated qualitatively.

### 2.7. IR Spectroscopy

**Figure 9:**
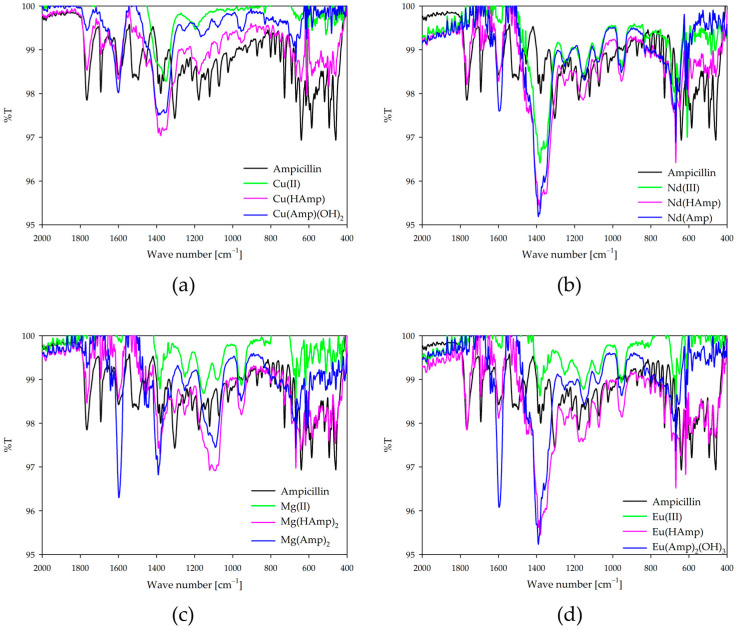
IR spectra of (**a**) Cu(II)/ampicillin (1:1 ratio); (**b**) Nd(II)/ampicillin (1:1 ratio); (**c**) Mg(II)/ampicillin (1:2 ratio); (**d**) Eu(II)/ampicillin (1:2 ratio).

Infrared spectra of the metal complexes with ampicillin confirm the involvement of the oxygen and nitrogen donor atoms in coordination. Spectra were recorded for dominant complexes and compared to free ampicillin and free metal ions under the same pH conditions (Figure 9). FTIR spectra were recorded for complex solutions prepared in D_2_O. To suppress background signals from the solvent, the instrument was calibrated using pure D_2_O as the reference background prior to each measurement. The observed changes in the intensity of the bands and their shifts suggest participation in the coordination of the carboxyl group, the carbonyl group of the amide, and the β-lactam ring, as well as the amine groups. Participation in the coordination of the carboxyl group is evidenced, among other things, by band differences in the 1390 cm^−1^ region for complexes compared to free ampicillin. This band corresponds to the symmetric stretching vibration (ν_S_ COO^−^) of the carboxylate group, which is known to occur in this region in aqueous solution environments [43,44]. In addition, confirmation of coordination through the donor oxygen atoms is provided by changes in the characteristic bands near 1685 cm^−1^, 1766 cm^−1^, and 3383 cm^−1^, which correspond, respectively, to coordination through the carbonyl group of the amide, the carbonyl group of the β-lactam ring, and the hydroxyl group (from the carbonyl group). The confirmation of the formation of coordination bonds with the central atom of the amino groups is provided by the changes found for the deformation vibrations of the amino groups (N-H, ~1505 cm^−1^) and the stretching vibrations (~3383 cm^−1^).

IR spectra of ampicillin (Figure S1) and all IR spectra for equimolar systems (Figure S2) and those with a two-fold excess of ampicillin (Figure S3) are included in the Supplementary Materials.

### 2.8. CD Spectroscopy

**Figure 10:**
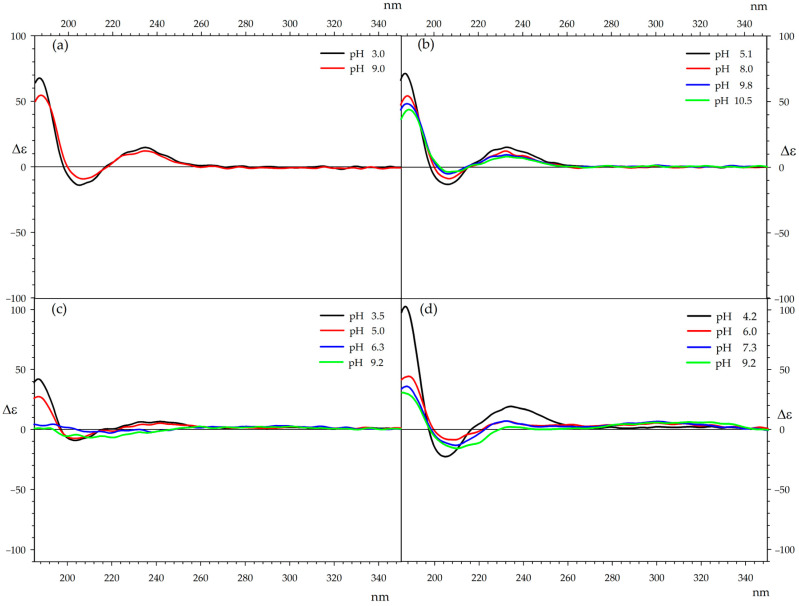
CD spectra of (**a**) ampicillin; (**b**) Co(II)/ampicillin (1:1 ratio); (**c**) Cu(II)/ampicillin (1:1 ratio); (**d**) Cu(II)/ampicillin (1:2 ratio).

To determine the conformational preferences of ampicillin complexes, circular dichroism (CD) spectra analysis was initiated by measuring the CD spectra of ampicillin at different pH levels. This approach was intended to distinguish the changes related to pH from those associated with interactions with metal cations when analyzing the CD spectra of ampicillin complexes. The CD spectra of ampicillin in water, measured at the same concentrations as the complex spectra, exhibit a long-wavelength Cotton effect (Δε) 14.8 at 235 nm, and two additional Cotton effects, Δε = −14.1 at 205 nm and Δε = 67.7 at 187 nm, at pH 3.0. At an alkaline pH 9.0, these effects are Δε = 12.0 at 235 nm, Δε = −9.3 at 207 nm, and Δε = 54.5 at 188 nm (Figure 10a). Thus, with increasing pH, a decrease of approximately 20% in the Cotton effects is observed, with no significant changes in their positions, indicating that the antibiotic’s conformation remains unchanged regardless of pH. The CD spectra of the ampicillin complexes were recorded at pH values previously determined by potentiometric titration. In all cases, the measurements were conducted for both equimolar complexes and those with a twofold excess of the antibiotic (Table 5, Table 6 and Table 7).

Al-Khodir’s group synthesized Amp complexes with iron(III), palladium(II), and gold(III) [45]. In the complexes with iron(III) and gold(III), coordination occurs through the amide carbonyl group, the β-lactam carbonyl group, the amino group, and the carboxylate group of Amp. In contrast, the palladium(II) complex is coordinated via the β-lactam carbonyl group and the carboxylate group. The Abbas group synthesized Schiff bases of Amp with acetylacetone, along with their complexes containing cobalt(II), nickel(II), copper(II), europium(III), and gadolinium(III) [46]. In these complexes, coordination occurs through the amide nitrogen atom, the β-lactam carbonyl group, and the amino group. The spatial structure of the studied compounds appears to be similar to that obtained by Abbas, which explains the results observed during circular dichroism (CD) measurements. Due to the relatively high rigidity of the ampicillin structure, the CD spectra of its complexes do not undergo significant changes. An example can be the CD spectra recorded over a wide pH range for cobalt complexes with ampicillin in a 1:1 molar ratio (Figure 10b).

When the pH changes, the amino group undergoes protonation in acidic conditions, while the carboxyl group ionizes in alkaline conditions. The aromatic chromophore is positioned away from the complexation sites, and its orientation relative to the rest of the molecule remains largely unchanged with pH variations.

The only deviation from the general trend is observed in the CD spectra of copper complexes. In the case of the Cu-Amp 1:1 complex under alkaline conditions, there is a reversal of the long-wavelength Cotton effect and a disappearance of the short-wavelength effect located around 188 nm (Figure 10c). Interestingly, in the system with a twofold excess of ampicillin, these effects are reduced by approximately half, suggesting that the second molecule does not participate in complexation in the same manner as the first molecule (Figure 10d). The changes observed in the CD spectra for copper complexes are reflected in the distribution diagrams. For alkaline solutions, a significant presence of Cu-antibiotic complexes is still observed, while, compared to other systems, the amount of metal in the form of hydroxide is relatively low.

All CD spectra for equimolar systems (Figure S4) and those with a two-fold excess of ampicillin (Figure S5) are included in the Supplementary Materials.

## 3. Materials and Methods

### 3.1. Materials

Metal nitrates (Mg(NO_3_)_2_·2H_2_O, Ca(NO_3_)_2_·4H_2_O, Sr(NO_3_)_2_, Co(NO_3_)_2_·6H_2_O, Ni(NO_3_)_2_·6H_2_O, Cu(NO_3_)_2_·3H_2_O, Nd(NO_3_)_3_·6H_2_O, Eu(NO_3_)_3_·5H_2_O, Tb(NO_3_)_3_·5H_2_O) were purchased from Merck (Darmstadt, Germany) (>99% purity) and used as supplied. Ampicillin, anhydrous basis (96.0–102.0%) obtained from Merck (Darmstadt, Germany), was used without further purification.

### 3.2. Equilibrium Studies

The equilibrium studies were obtained by potentiometric measurements, which were performed using a Titrando 905 Metrohm equipped with an autoburette with an electrode Metrohm 6.0233.100 (Metrohm AG, Herisau, Switzerland). This equipment was calibrated in terms of hydrogen ion concentration prior to each series of titrations [37,38,39,47,48,49].

Before each series of measurements, the glass electrode (Metrohm AG, Herisau, Switzerland) was calibrated daily using two standard buffer solutions of pH 4.00 and pH 9.00, and the water ionization constant (pK_w_ = 13.77 at 20 °C, I = 0.1 mol·dm^−3^) was determined in situ by titrating acidified KNO_3_ solutions. The conditions under which the measurements were taken were constant and clearly defined: temperature 20 ± 1 °C, constant ionic strength of 0.1 mol·dm^−3^ (KNO_3_), neutral gas atmosphere (helium—Ultra High Purity 5.0; Linde Gaz, Krakow, Poland), and a CO_2_-free NaOH titrant. Titrations used 30 mL samples and burette increments of 0.004 mL of base at a concentration of approximately 0.2 mol·dm^−3^. Furthermore, the concentration of metal ions was 0.001 mol·dm^−3^, and the metal:ligand ratio was 1:1 and 1:2 in binary systems.

Calculations were performed using the HYPERQUAD 2008 program (Hyperquad Limited, Leeds, UK) to determine the overall stability constants (log*β*) and the equilibrium constants of formation (log*K_e_*) of the complexes. On the basis of the calculations obtained from the program, models of the formation of complexes in the studied systems were proposed. To verify the precision of the assumed model, the Hamilton test and the chi-squared test [47,48,49] were used to examine the standard deviation values, and the experimental curve converged with the model. Additionally, the following equilibria were evaluated as stability constants of *o*M + *p*L + *q*H ⇆ M*o*L*p*H*q* (where M = metal ion, L = ligand), and calculated using the following equation:

(1) $$\beta =\frac{M_{o}L_{p}H_{q}}{{\left[M\right]}^{o}{\left[L\right]}^{p}{\left[H\right]}^{q}}$$

The hydrolysis constants of the metal ions were included in the computer analysis of the potentiometric data. These constants were taken from our previous article, and their values were the ionic product of water was p*K*_w_ = 13.77 [38,39,47,48]. The HySS (Hyperquad Simulation and Speciation) program was used to generate distribution diagrams of particular forms [37].

### 3.3. UV–Vis Spectroscopy

The UV–Vis absorption spectra were collected at room temperature using Evolution 300 UV–Vis ThermoFisher Scientific equipment (Thermo Electron Scientific Instruments LLC, Madison, WI, USA) with a xenon lamp and a Plastibrand PMMA cell (Brand, Wertheim, Germany) with a path length of 1 cm. Measurements covered wavelengths ranging from 340 to 1000 nm, while the concentration of metal ions was maintained at 0.001 mol·dm^−3^, with metal-to-ligand molar ratios of 1:1 and 1:2. Measurements were performed at pH values selected based on speciation diagrams, corresponding to conditions under which the highest proportion of the metal ion is involved in complex formation.

### 3.4. EPR Spectroscopy

EPR measurements were carried out at X-band frequency (ν = 8.9 GHz) and at a temperature of −196 °C (the temperature of liquid nitrogen) using glass capillary tubes with a volume of 130 µm^3^, and the data were recorded on an SE/X 2457 Radiopan spectrometer (Radiopan, Poznan, Poland). This study focused on copper ion systems with a metal ion concentration of 0.005 mol·dm^−3^ in a water:glycol mixture (3:1), examining both 1:1 and 1:2 metal to ligand ratios. All experimental spectra were simulated using the EPRsim32 program (version 0.3 alpha, Kraków, Poland) [50]. Measurements were performed at pH values selected based on speciation diagrams, corresponding to conditions under which the highest proportion of the metal ion is involved in complex formation.

### 3.5. Luminescence Spectroscopy

The luminescence studies were recorded on an RF−6000 spectrofluorophotometer (Shimadzu, Kyoto, Japan) using 5/5 nm slit widths. The concentration of metal ions was 0.001 mol·dm^−3^. The samples for europium ions were excited at 395 nm, and those for terbium ions were excited at 370 nm. The absorbance of the solutions at the excitation wavelength was <0.2 absorbance units. The samples were prepared using ultra-high-quality deionized and purified water from a Simplicity Ultrapure Water System (Millipore, Darmstadt, Germany). Measurements were performed at pH values selected based on speciation diagrams, corresponding to conditions under which the highest proportion of the metal ion is involved in complex formation.

### 3.6. Infrared Spectroscopy (FT-IR)

Infrared studies were recorded on an FT-IR INVENIO R spectrophotometer (Bruker, Bremen, Germany). Samples were prepared by dissolving the relevant ligand species (ampicillin and appropriate metal nitrates) in D_2_O. The metal concentration for the IR studies was 0.1 mol·dm^−3^, with metal:ligand molar ratios of 1:1 and 1:2; the concentration was higher to ensure sufficient absorbance in transmission. The pH values were adjusted by the addition of NaOD or DCl. The pH values were corrected according to the formula pD = pH meter reading + 0.4 [49]. Measurements were performed at pH values selected based on speciation diagrams, corresponding to conditions under which the highest proportion of the metal ion is involved in complex formation.

### 3.7. CD Spectroscopy

The circular dichroism (CD) studies were recorded on the J810 spectropolarimeter (JASCO, Tokyo, Japan) in the range of 180–400 nm in water solutions. Spectra were recorded at ambient temperature and accumulated with 4 scans, both for free ampicillin and ampicillin complexes. The measurements were made in nitrogen gas (flow 10 L/min) with an optical path length of 0.5 mm. The concentrations of the metals in solutions were 1 × 10^−4^ mol·dm^−3^. Measurements were performed at pH values selected based on speciation diagrams, corresponding to conditions under which the highest proportion of the metal ion is involved in complex formation.

## 4. Conclusions

The formation of binary complexes in binary systems of ampicillin with selected metal ions has been thoroughly investigated and established. In the equimolar system, the researchers found that only complex forms with one coordinated ampicillin molecule were present. However, in the case of a twofold excess of the antibiotics, all studied metals—except for Ca(II) and Sr(II), which remain as in equimolar system species—even at high ligand concentrations, form complexes, with two ligand molecules located in the coordination sphere. As the pH of the system increased, a progression was observed from protonated forms initially, followed by the formation of simple complexes, and finally hydroxy complexes. The complex formation and the inner coordination sphere were confirmed through the use of various spectroscopic methods, including infrared, UV–Vis, and luminescence measurements. The studies were conducted at different pH conditions to validate the changes in the internal coordination sphere caused by the attachment of subsequent functional groups to the central atom. Infrared spectroscopy studies confirmed the participation of the antibiotic functional groups in the formation of coordination bonds with the selected metal ions. UV–Vis studies revealed changes in absorption and shifts in absorbance during the formation of complexes with the oxygen donor atom at lower pH values. Luminescence measurements showed a decrease in the emission of europium ions, while a shift and enhancement in the ^5^D_4_ -^7^F_5_ band were observed for terbium ions. Furthermore, circular dichroism studies demonstrated the conformational stability of ampicillin throughout the entire studied range.

## Figures and Tables

**Table 1:** The overall stability constants (log*β*) and the equilibrium constants of formation (log*K_e_*) of the complexes formed in the studied systems with ampicillin and s-block elements (standard deviations are given in parentheses).

Species		Mg(II)		Ca(II)		Sr(II)	
		log*β*	log*K_e_*	log*β*	log*K_e_*	log*β*	log*K_e_*
**1:1**	M(HAmp)	9.85(5)	2.75	10.00(2)	2.90	9.99(1)	2.89
	M(Amp)	3.60(2)	3.60	-	-	2.60(7)	2.60
	M(Amp)(OH)	-	-	-	-	−8.56(5)	2.60
**1:2**	M(HAmp)	-	-	10.00(2)	2.90	9.99(1)	2.89
	M(HAmp)(HAmp)	20.91(4)	6.72	-	-	-	-
	M(HAmp)(Amp)	14.23(5)	7.13	-	-	-	-
	M(Amp)_2_	6.96(4)	3.36	-	-	-	-

**Table 2:** The overall stability constants (log*β*) and the equilibrium constants of formation (log*K_e_*) of the complexes formed in the studied systems with ampicillin and d-block elements (standard deviations are given in parentheses).

Species		Co(II)		Ni(II)		Cu(II)	
		log*β*	log*K_e_*	log*β*	log*K_e_*	log*β*	log*K_e_*
**1:1**	M(HAmp)	9.99(6)	2.90	10.91(2)	3.82	10.36(4)	3.26
	M(Amp)	3.12(6)	3.12	3.86(1)	3.86	5.23(5)	5.23
	M(Amp)(OH)	-	-	−6.21(1)	3.70	0.29(2)	8.83
	M(Amp)(OH)_2_	−14.88(8)	12.66	-	-	−7.48(3)	5.99
	M(Amp)(OH)_3_	−25.33(4)	3.32	-	-	−18.67(4)	2.58
**1:2**	M(HAmp)(HAmp)	20.49(4)	6.30	19.99(3)	5.80	20.54(2)	6.35
	M(HAmp)(Amp)	13.96(4)	6.86	13.60(3)	6.50	15.75(4)	8.66
	M(Amp)_2_	6.22(5)	3.10	6.27(2)	2.41	10.89(2)	5.65
	M(Amp)_2_(OH)	-	-	-	-	4.06(2)	6.95
	M(Amp)_2_(OH)_2_	−11.84(3)	9.47	-	-	−3.85(2)	5.85

**Table 3:** The overall stability constants (log*β*) and the equilibrium constants of formation (log*K_e_*) of the complexes formed in the studied systems with ampicillin and f-block elements (standard deviations are given in parentheses).

Species		Nd(III)		Eu(III)		Tb(III)	
		log*β*	log*K_e_*	log*β*	log*K_e_*	log*β*	log*K_e_*
**1:1**	M(HAmp)	10.82(2)	3.72	10.76(4)	3.66	10.45(2)	3.18
	M(Amp)	3.42(2)	3.42	-	-	-	-
	M(Amp)(OH)_3_	-	-	−21.11(4)	20.20	−21.06(1)	20.24
	M_2_(Amp)	-	-	7.47(3)	7.47	-	-
**1:2**	M(HAmp)	10.82(2)	3.72	10.76(4)	3.66	10.45(2)	3.35
	M(Amp)	3.42(2)	3.42	-	-	-	-
	M(HAmp)(Amp)	-	-	13.45(5)	13.45	-	-
	M(Amp)_2_	-	-	6.50(6)	6.50	-	-
	M(Amp)(OH)_3_	-	-	-	-	−21.06(1)	20.24
	M(Amp)_2_(OH)_3_	-	-	−16.81(5)	4.22	-	-
	M(Amp)_2_(OH)_4_	-	-	−26.77(4)	3.81	-	-

**Table 4:** Spectral parameters of binary complexes formed in systems of d-electron metal ions with ampicillin.

Species			pH	A	λ_max_ (nm)	ε (dm^3^·mol^−1^·cm^−1^)
**1:1**	Co(II)	M(HAmp)	5.3	0.0096	517	9.6
		M(Amp)	7.9	0.0084	510	8.4
	Ni(II)	M(HAmp)	5.3	0.0056 0.0023	670 905	5.6 2.3
		M(Amp)	8.1	0.0036 0.0016	675 900	3.6 1.6
	Cu(II)	M(HAmp)	3.6	0.018	775	18
		M(Amp)	4.9	0.055	710	55
		M(Amp)(OH)	6.4	0.13	610	130
**1:2**	Co(II)	M(HAmp)(HAmp)	5.1	0.012	510	12
		M(HAmp)(Amp)	7.0	0.011	510	11
		M(Amp)_2_	8.1	0.020	490	20
	Ni(II)	M(HAmp)(HAmp)	5.2	0.0052 0.0022	700 890	5.2 2.2
		M(HAmp)(Amp)	6.1	0.0045 0.0021	710 894	4.5 2.1
		M(Amp)_2_	8.7	0.0031 0.0019	644 900	3.1 1.9
	Cu(II)	M(HAmp)(Amp)	4.2	0.044	725	44
		M(Amp)_2_	5.6	0.13	630	130

**Table 5:** Values of Cotton effects for the systems with ampicillin and s-block elements.

Metal/Ampicillin (1:1 Ratio)						Metal/Ampicillin (1:2 Ratio)					
Mg(II)		Ca(II)		Sr(II)		Mg(II)		Ca(II)		Sr(II)	
pH	∆ε (nm)	pH	∆ε (nm)	pH	∆ε (nm)	pH	∆ε (nm)	pH	∆ε (nm)	pH	∆ε (nm)
4.3	13.7 (233)	5.0	12.0 (233)	5.0	12.5 (232)	4.5	25.6 (233)	5.0	26.4 (233)	5.0	25.0 (233)
	−12.1 (207)		−11.3 (206)		−12.2 (207)		−23.5 (208)		−23.8 (207)		−23.1 (207)
	64.8 (187)		59.5 (187)		59.6 (187)		126.7 (187)		127.0 (187)		125.5 (187)
8.8	11.3 (234)			9.0	11.8 (233)	7.0	25.1 (234)				
	−8.9 (206)				−7.6 (206)		−19.6 (207)				
	49.7 (188)				46.0 (189)		115 (187)				
						9.0	24.1 (233)				
							−15.3 (207)				
							102.1 (188)				

**Table 6:** Values of Cotton effects for the systems with ampicillin and d-block elements.

Metal/Ampicillin (1:1 Ratio)						Metal/Ampicillin (1:2 Ratio)					
Co(II)		Ni(II)		Cu(II)		Co(II)		Ni(II)		Cu(II)	
pH	∆ε (nm)	pH	∆ε (nm)	pH	∆ε (nm)	pH	∆ε (nm)	pH	∆ε (nm)	pH	∆ε (nm)
5.1	15.2 (233)	4.5	15.7 (233)	3.5	6.5 (242)	5.0	26.8 (233)	4.4	27.0 (233)	4.2	19.2 (235)
	−13.0 (207)		−15.0 (207)		−9.2 (204)		−23.7 (207)		−21.0 (206)		−22.7 (205)
	71.4 (187)		77.6 (187)		42.0 (187)		126.1 (187)		128.2 (187)		102.7 (187)
8.0	12.3 (233)	8.3	15.5 (233)	5.0	5.1 (242)	7.1	23.8 (233)	6.8	25.7 (232)	6.0	6.9 (232)
	−8.7 (207)		−7.3 (207)		−7.5 (203)		−19.3 (206)		−19.4 (206)		−8.8 (209)
	54.4 (188)		57.2 (188)		27.3 (187)		112.4 (188)		118.3 (187)		44.3 (189)
9.8	9.4 (233)			6.3	3.0 (294)	8.5	19.0 (233)	8.2	23.7 (233)	7.3	6.6 (305) 7.0 (232)
	−5.0 (207)				−2.3 (238) −3.1 (220)		−11.3 (207)		−14.1 (207)		−13.3 (210)
	48.3 (188)				4.3 (194)		88.7 (188)		98.7 (188)		36.0 (188)
10.5	8.2 (233)			9.2	2.6 (301) 2.6 (260)	10.0	17.9 (233)			9.2	6.2 (314) 2.2 (234)
	−3.4 (207)				−6.5 (221) −7.0 (210) −5.5 (200)		−7.7 (208)				−15.7 (210)
	44.0 (189)						84.1 (189)				30.9 (186)

**Table 7:** Values of Cotton effects for the systems with ampicillin and f-block elements.

Metal/Ampicillin (1:1 Ratio)						Metal/Ampicillin (1:2 Ratio)					
Nd(III)		Eu(III)		Tb(III)		Nd(III)		Eu(III)		Tb(III)	
pH	∆ε (nm)	pH	∆ε (nm)	pH	∆ε (nm)	pH	∆ε (nm)	pH	∆ε (nm)	pH	∆ε (nm)
5.0	10.8 (236)	5.0	13.3 (236)	5.0	15.9 (236)	5.0	25.8 (235)	2.4	23.3 (232)	5.0	28.4 (235)
	−9.8 (204)		−13.5 (207)		−14.0 (206)		−24.8 (206)		−27.9 (206)		−26.0 (206)
	57.1 (187)		74.3 (187)		80.2 (187)		131.6 (187)		123.3 (187)		137.3 (188)
7.7	10.7 (235)	7.5	13.5 (235)	9.2	13.8 (236)	8.1	22.9 (235)	5.0	26.7 (233)	9.2	24.7 (235)
	−8.4 (205)		−10.6 (205)		−11.1 (206)		−17.0 (207)		−23.1 (206)		−19.3 (206)
	48.6 (187)		66.4 (187)		60.4 (188)		104.0 (188)		127.1 (187)		108.7 (189)
		10.0	11.9 (236)					7.4	23.9 (235)		
			−8.8 (206)						−19.2 (207)		
			54.9 (188)						112.9 (188)		
								9.0	22.9 (234)		
									−16.2 (207)		
									101.4 (188)		
								10.0	20.9 (235)		
									−15.7 (207)		
									91.2 (189)		

## Data Availability

All data generated or analyzed during this study are included in this published article (and its Supplementary Information File).

## Supplementary Materials

The following supporting information can be downloaded at https://www.mdpi.com/article/10.3390/ijms26157605/s1.

## Abbreviations

The following abbreviations are used in this manuscript:

Amp	Ampicillin
