# Supplementary material for: Insights into Complex Compounds of Ampicillin: Potentiometric and Spectroscopic Studies
Source: Int J Mol Sci. 2025 Aug 6;26(15):7605. doi: 10.3390/ijms26157605 (PMC12347571; doi:10.3390/ijms26157605)
Supplement: Supplementary file 1 [file ijms-26-07605-s001.zip › ijms-3739687-supplementary.pdf]

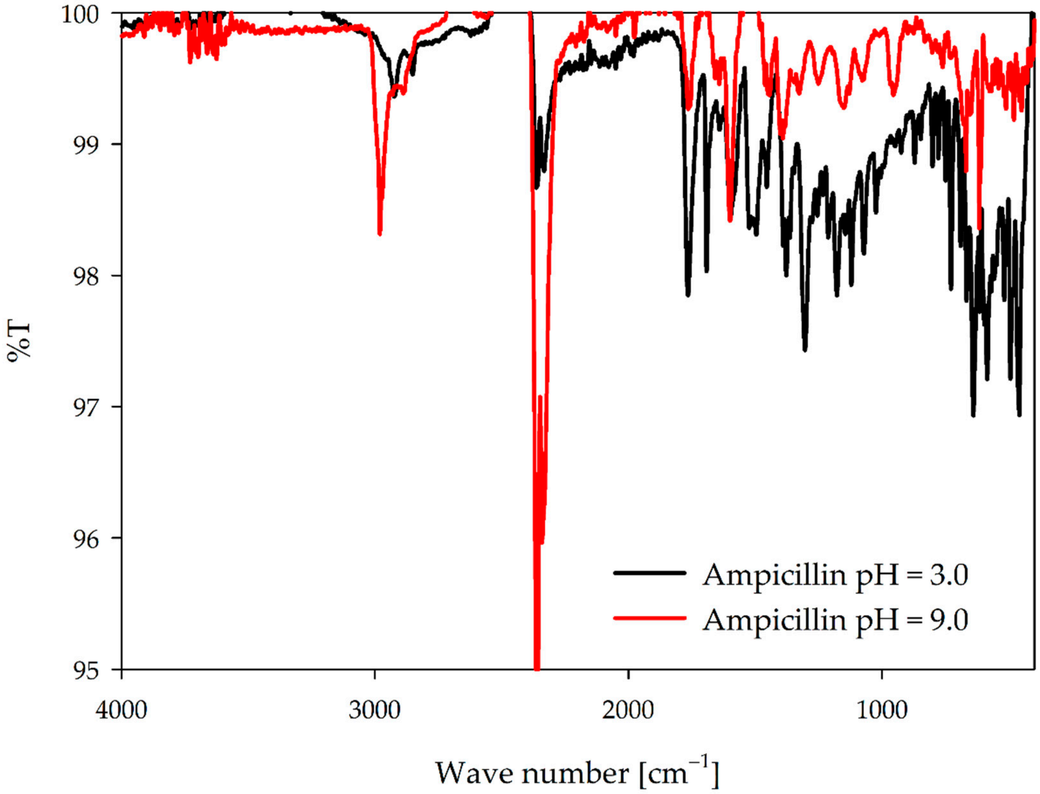

Figure S1. IR spectra of ampicillin.

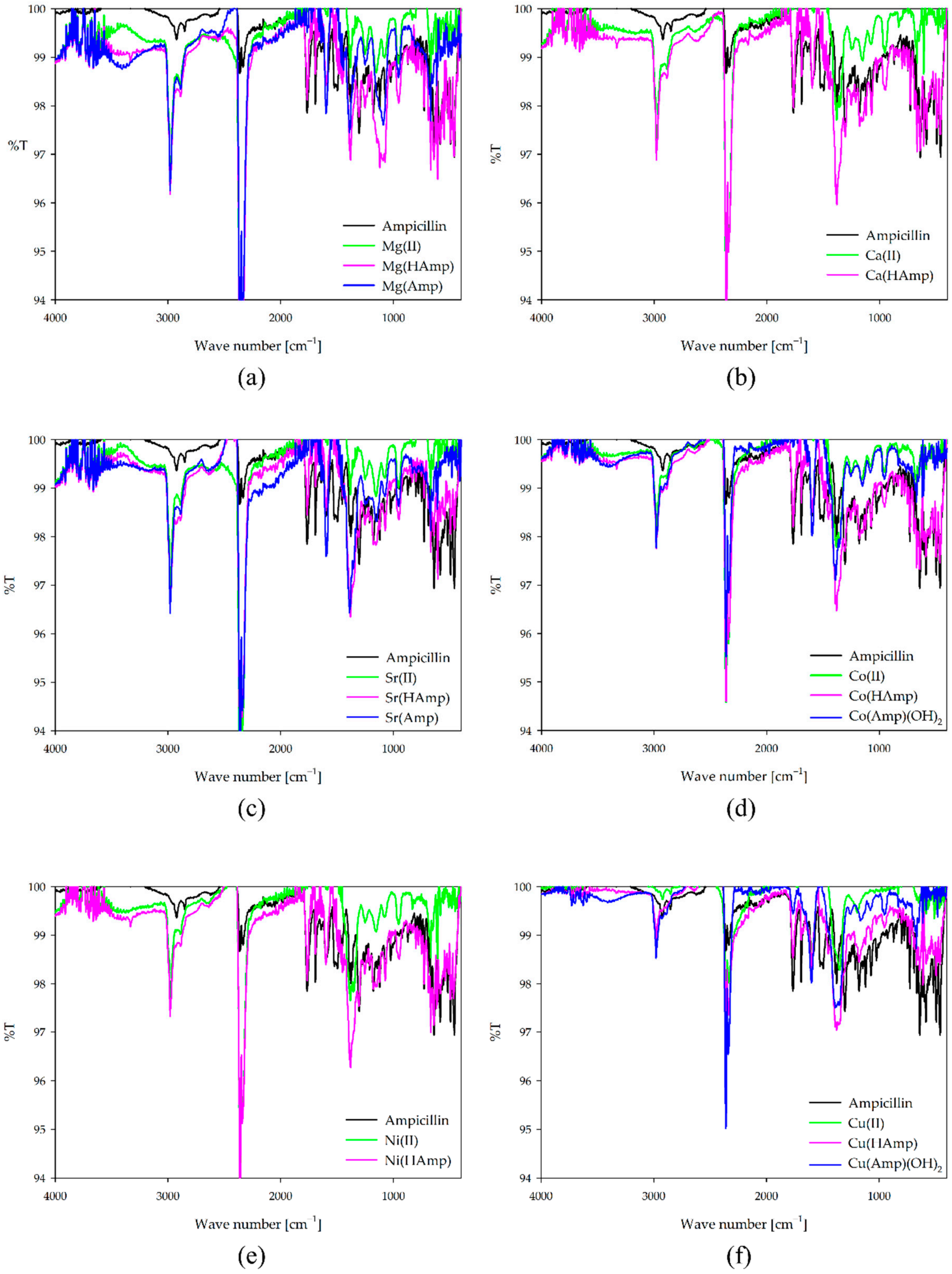

%T
Wave number [cm−1]
Ampicillin
Mg(II)
Mg(HAmp)
Mg(Amp)
(a)
Ampicillin
Ca(II)
Ca(HAmp)
(b)
Ampicillin
Sr(II)
Sr(HAmp)
Sr(Amp)
(c)
Ampicillin
Co(II)
Co(HAmp)
Co(Amp)(OH)2
(d)
Ampicillin
Ni(II)
Ni(HAmp)
(e)
Ampicillin
Cu(II)
Cu(HAmp)
Cu(Amp)(OH)2
(f)

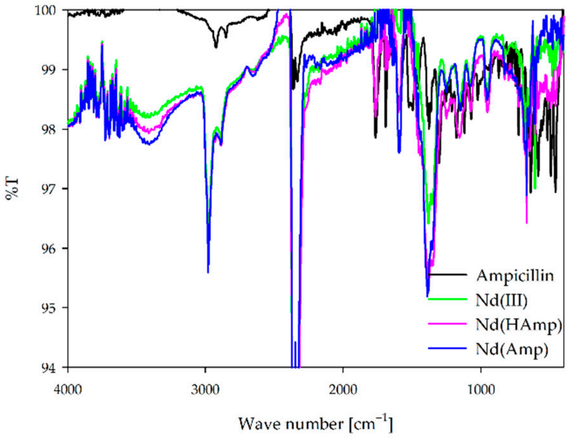

(g)

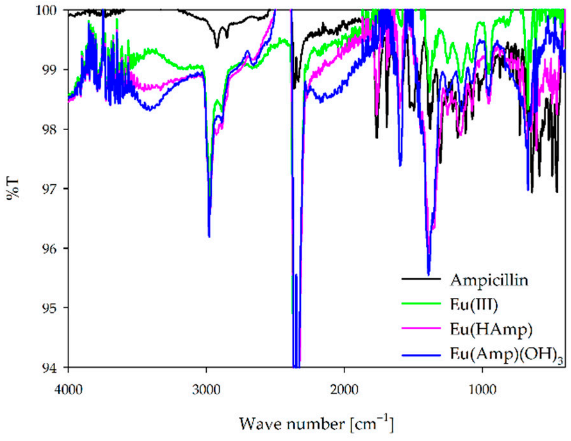

(h)

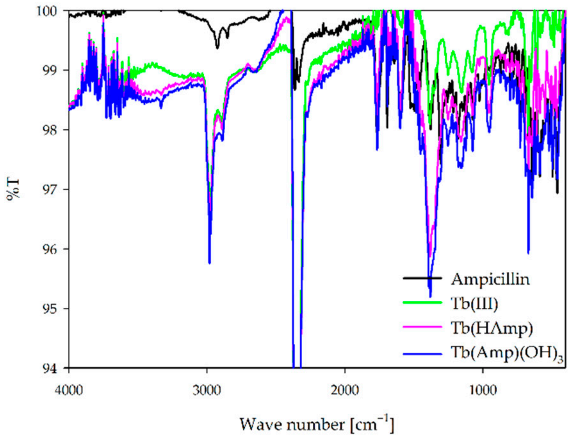

(i)

Figure S2. IR spectra in equimolar system of: (a) Mg(II)/ampicillin; (b) Ca(II)/ampicillin; (c) Sr(II)/ampicillin; (d) Co(II)/ampicillin; (e) Ni(II)/ampicillin; (f) Cu(II)/ampicillin; (g) Nd(III)/ampicillin; (h) Eu(III)/ampicillin; (i) Tb(III)/ampicillin.

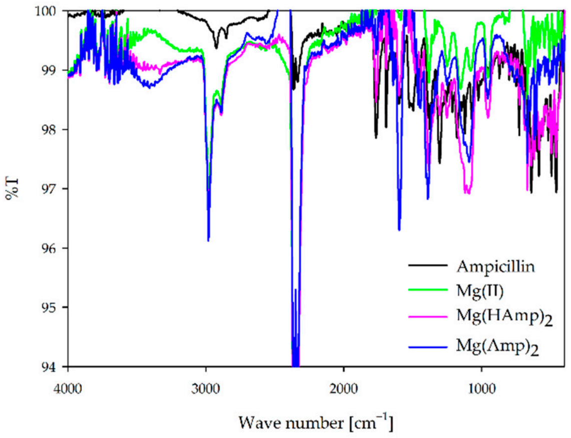
Ampicillin
Mg(II)
Mg(HAmp)2
Mg(Amp)2
%T
Wave number [cm−1]

(a)

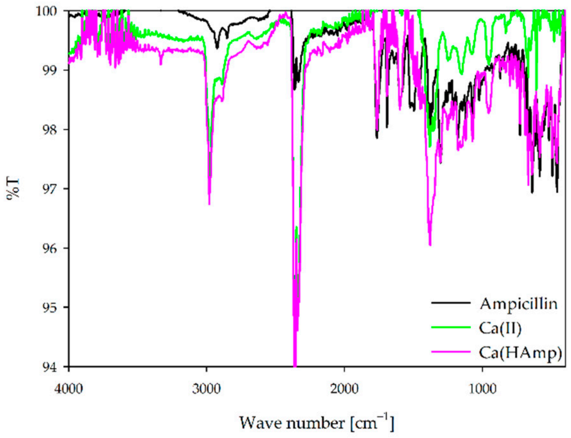
Ampicillin
Ca(II)
Ca(HAmp)
%T
Wave number [cm−1]

(b)

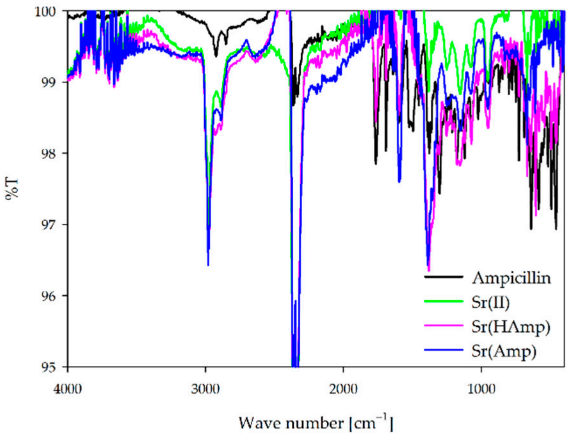
Ampicillin
Sr(II)
Sr(HAmp)
Sr(Amp)
%T
Wave number [cm−1]

(c)

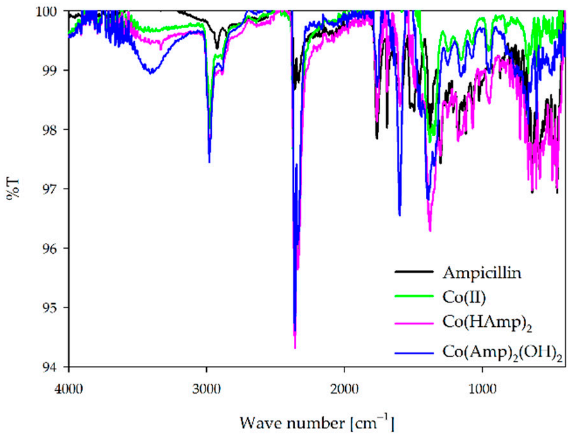
Ampicillin
Co(II)
Co(HAmp)2
Co(Amp)2(OH)2
%T
Wave number [cm−1]

(d)

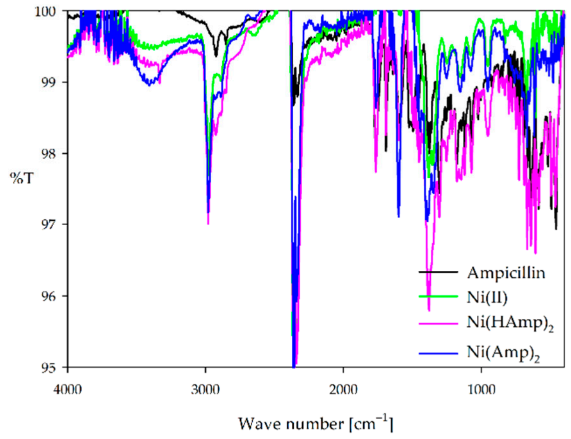
Ampicillin
Ni(II)
Ni(HAmp)2
Ni(Amp)2
%T
Wave number [cm−1]

(e)

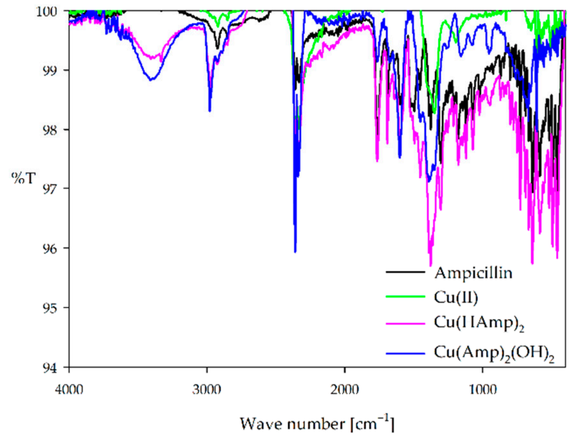
Ampicillin
Cu(II)
Cu(HAmp)2
Cu(Amp)2(OH)2
%T
Wave number [cm−1]

(f)

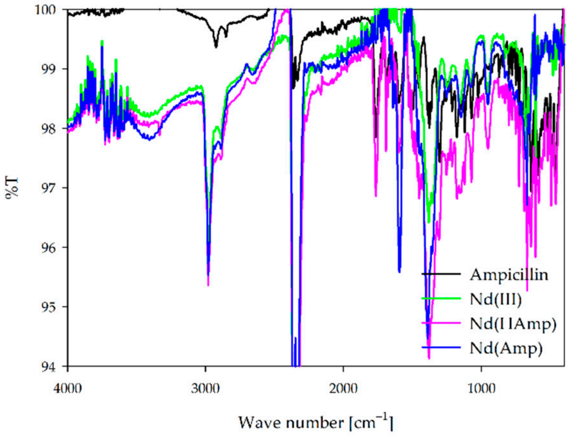

(g)

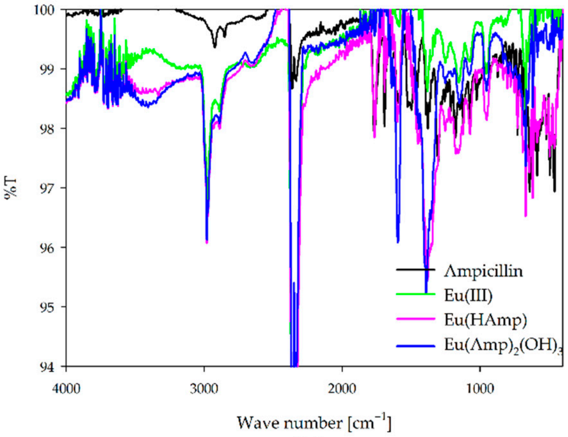

(h)

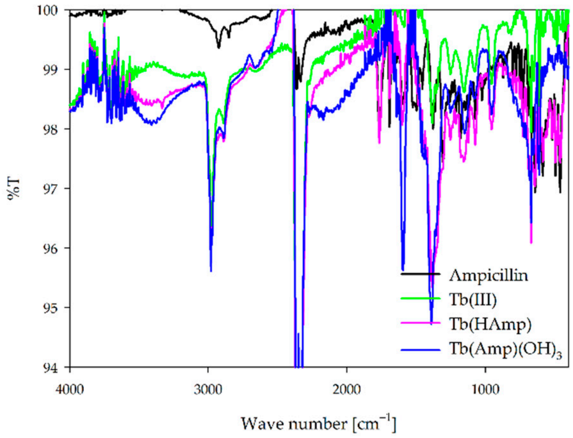

(i)

Figure S3. IR spectra in a system with a two-fold excess of antibiotic of: (a) Mg(II)/ampicillin; (b) Ca(II)/ampicillin; (c) Sr(II)/ampicillin; (d) Co(II)/ampicillin; (e) Ni(II)/ampicillin; (f) Cu(II)/ampicillin; (g) Nd(III)/ampicillin; (h) Eu(III)/ampicillin; (i) Tb(III)/ampicillin.

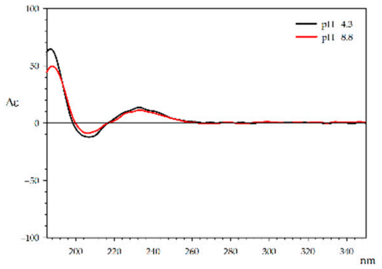

(a)

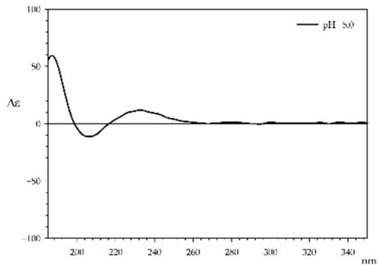

(b)

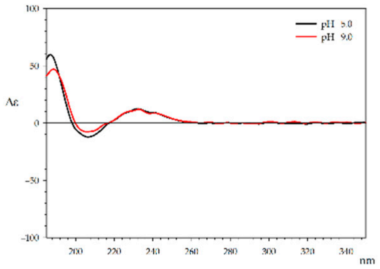

(c)

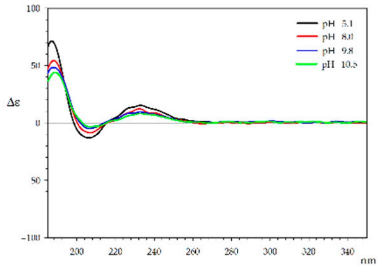

(d)

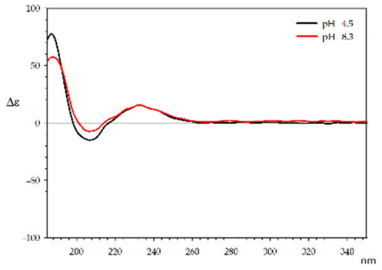

(e)

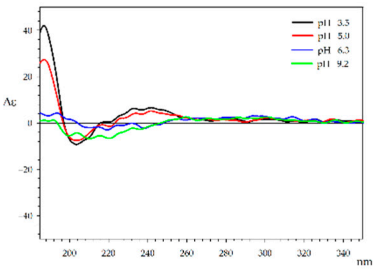

(f)

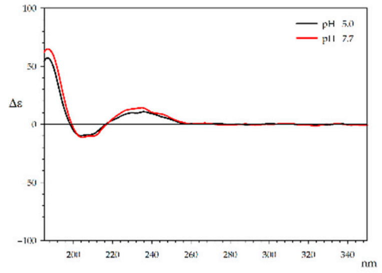

(g)

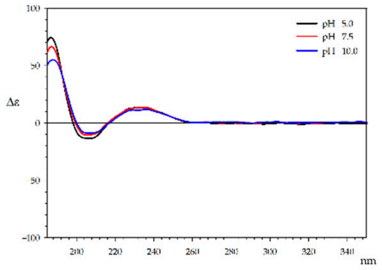

(h)

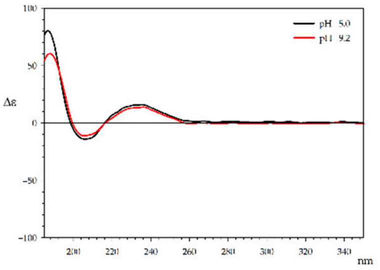

(i)

Figure S4. CD spectra in equimolar system of: (a) Mg(II)/ampicillin; (b) Ca(II)/ampicillin; (c) Sr(II)/ampicillin; (d) Co(II)/ampicillin; (e) Ni(II)/ampicillin; (f) Cu(II)/ampicillin; (g) Nd(III)/ampicillin; (h) Eu(III)/ampicillin; (i) Tb(III)/ampicillin.

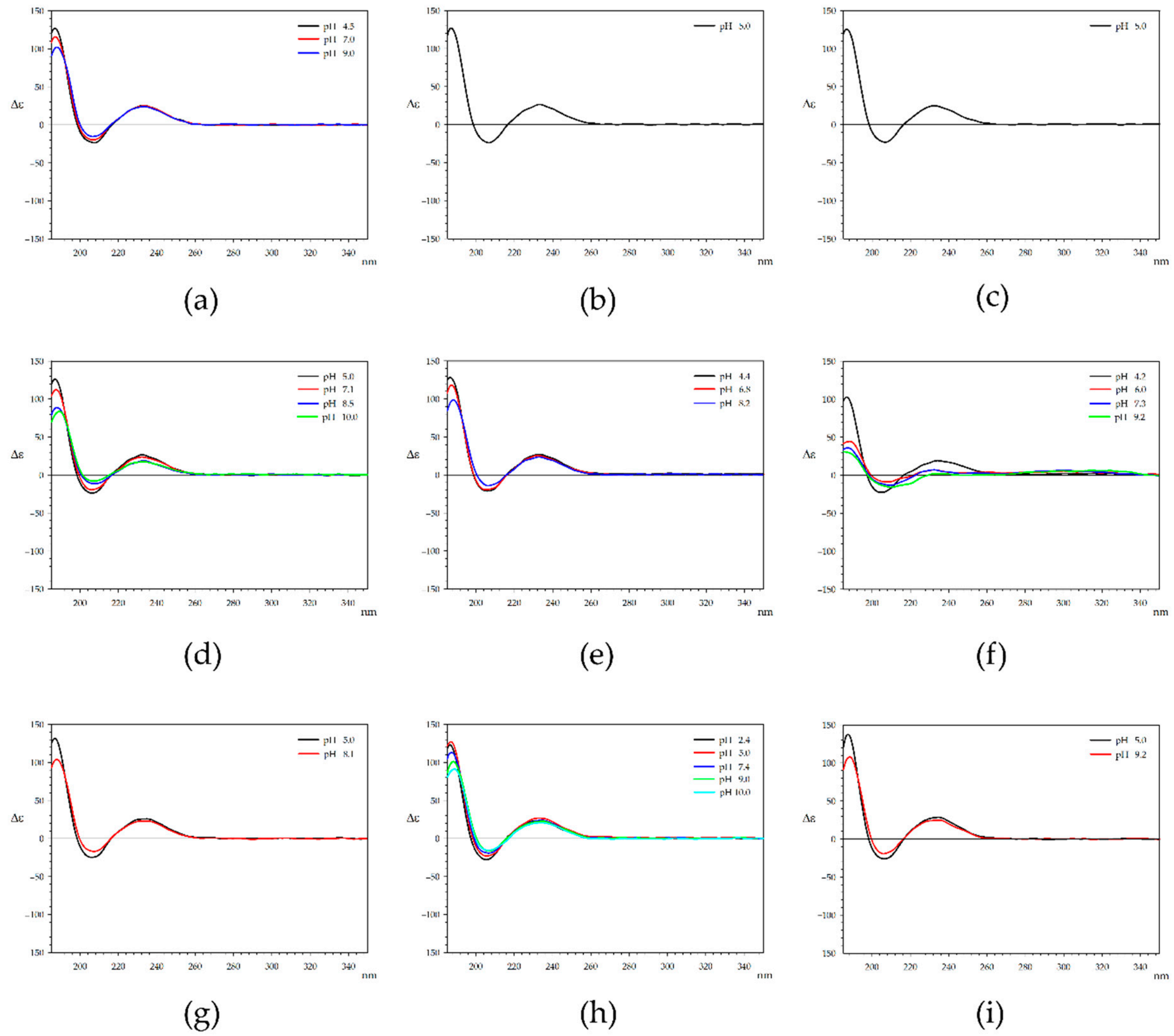

Figure S5. CD spectra in a system with a two-fold excess of antibiotic of: (a) Mg(II)/ampicillin; (b) Ca(II)/ampicillin; (c) Sr(II)/ampicillin; (d) Co(II)/ampicillin; (e) Ni(II)/ampicillin; (f) Cu(II)/ampicillin; (g) Nd(III)/ampicillin; (h) Eu(III)/ampicillin; (i) Tb(III)/ampicillin.
